# Evaluation of the Rational Analgesic use in elderly adults: A cross-sectional study

**DOI:** 10.12669/pjms.36.5.2331

**Published:** 2020

**Authors:** Melike Mercan Baspınar, Okcan Basat

**Affiliations:** 1 Dr. Melike Mercan Başpinar, Department of Family Medicine, University of Health Sciences, Gaziosmanpaşa Training and Research Hospital, Istanbul, Turkey; 2 Dr. Okcan Basat, Department of Family Medicine, University of Health Sciences, Gaziosmanpaşa Training and Research Hospital, Istanbul, Turkey

**Keywords:** Analgesic, Beers, family medicine, pain, STOPP/START

## Abstract

**Objectives:**

To assess inappropriate analgesic use (IAU) by comparison of STOPP/START Version-2 and Beers 2019 criteria.

**Methods:**

This is a cross-sectional study of 331 elderly patients admitted to family medicine clinics at a tertiary hospital between February and July 2018. Data were collected from face-to-face surveys, including informed patient consent and electronic drug monitoring databases.

**Results:**

The presence of IAU by STOPP version2 was higher than the Beers criteria (19.6%, 14.5%, respectively; P<0.04; Z= –2.5) with a moderate agreement (Kappa= 0.458). The number of drugs and pain score were predictors of IAU. The most commonly caused IAU was diclofenac, although naproxen was the most used analgesic. Almost 39% of diclofenac use, 18.5% of naproxen use, and 33% of etodolac use were IAU. Most commonly, IAU reasons were; (i) NSAID use in hearth failure (Beers) (ii) NSAID use with an antiplatelet agent(s) without PPI (STOPP).

**Conclusions:**

The difference between criteria in terms of IAU was significant in favor of STOPP V2.

## INTRODUCTION

When a pain appeared in the knee or back through aging, people begin to understand how old they are. Another way to define aging maybe by looking at the numbers of drug boxes. Elderly patients having a history of multiple drug use and suffering from pain are a problem for doctors because of the need for painkillers.

The meaning of pain will differ among patients depending on how pain affects their lives.^1^ Especially, joint pain has been shown to increase with age.^2^ Elderly adults comprise the group most likely to suffer both acute and chronic painful diseases, to have several diseases, and to take multiple medications.^3^ Although polypharmacy is a significant problem, some criteria lead to detect rational drug use and prescription.

The Beers Criteria identifies potentially inappropriate medications for better therapeutic regimens. As a result of the need to alert healthcare providers to the potential harms of specific medications, Beers Criteria was published in 1991 and recently updated in 2019.^4^,^5^

STOPP ( Screening Tool of Older Person’s Prescription ) Version-2 /START (Screening Tool to Alert doctor to Right Treatment) criteria have been expanded and updated in 2015 recently to minimize inappropriate prescribing in elderly patients. These criteria are based on an up-to-date literature review and consensus validation among a European panel of experts.^6^

Both criteria were selected because they are the most used and cited in studies on inappropriate drug prescribing in elderly patients.^4^,^7^,^10^ To our knowledge, no study was done about inappropriate analgesic use and pain medication by comparing the success of the Beers 2019 and STOPP/START Version-2 criteria.

We hypothesized; (i) Patients 65 years and older do not use rational analgesics (ii) There might be significant differences in the detection of IAU according to Beers and STOPP criteria (iii) There might be low awareness of painkillers and side effects, as well as the over or underuse of analgesics in pain treatment, especially about opioid group analgesics.

We aimed; (i) To assess IAU and related factors in elderly adults compared with Beers and STOPP criteria (ii) To search the presence of pain and the awareness of NSAID and opioid analgesics.

## METHODS

The study was performed by a cross-sectional evaluation of 331 older patients (≥aged 65 years) who were admitted to the family medicine outpatient clinics of a tertiary training and research hospital between February and July 2018. Data were collected from face-to-face surveys, including informed patient consent and electronic drug monitoring databases. Ethical permission to conduct this study was obtained from the Local Ethics Committee (Ref.No: 125, dated 24/01/2018 and updated 13/03/2019). The study was conducted in compliance with the Declaration of Helsinki.

Patients who were prescribed analgesic by the Algology clinic for chronic pain management, patients diagnosed with advanced-stage dementia or Alzheimer’s disease, and patients receiving active cancer treatment were excluded. All medications that participants had taken within the last two weeks were taken into consideration.

### Determination of the list for IAU

Beers’ 2015 criteria were available when the study was started, and the 2019 update was published during the analysis of the study. Therefore we examined the IAU using the American Geriatric Society’s Beers Criteria 2019 and STOPP /START Version-2 criteria. We assessed criteria for i) Appropriateness of analgesic drug use ii) Another drug- Analgesic drug interaction iii) Analgesic drug- Disease interaction iv) Therapeutic duplications of analgesic drugs v) Underuse of analgesic drugs vi) Analgesic use with caution

### Measurement and medication of pain

The pain was measured using the visual analog scale for pain (VAS). The pain was defined according to the World Health Organization (WHO) scale, which was a 0–10 numeric rating scale.^11^ Pain medication was classified according to the American Geriatric Society (AGS) guidelines,^12^ which classify recommended drugs for pain as follows nonopioid, opioid, adjuvant drug, and other drugs.

### Data Analysis

Descriptive statistics were used to measure the frequency, mean, and standard deviation of all variables. Covariates were analyzed using the Mann-Whitney U test and the Chi-square test; correlations were done by the Spearman test; furthermore, logistic regression analyses were used to evaluate the risk factors affecting IAU. According to Beers and STOPP criteria, IAU numbers were compared with the Wilcoxon test. Agreement in between Beers 2019 and STOPP Version-2 was checked with the Kappa test. Data were analyzed with the NCSS 10 (2015. Kaysville, Utah, USA) program.

## RESULTS

Out of 331 patients aged between 65 and 97 years (M age = 73.65; SD age = 7.03), 176 (53.2%) were female and 155 (46.8%) were male. The mean number of chronic diseases was 3.30±1.51 (Median = 3). The distribution of chronic diseases was hypertension 22.7% (n: 248), diabetes 11.3% (n: 124), coronary artery disease 7.9% (n: 86), chronic renal failure 7.5% (n: 82), respectively. The mean number of drug consumption was 6,87±2.76 (Maximum = 15), and the mean number of the analgesic drug was 0.66±0.41 (Maximum = 3). Polypharmacy was defined as using greater than or equal to five medications, and the prevalence of polypharmacy among our patients was 87.3%.

The association of IAU with demographic data and pain medication for Beers 2019 and STOPP/START criteria Version-2 is shown in Table-I. There was a statistically significant difference between the education level (STOPP P-value:0.025) and polypharmacy (+/-) groups (STOPP P-value: 0.03 START P-value:0.03) in terms of IAU. There was no IAU in high-level education group (high school and university) according to STOPP criteria. The underuse (START criteria) of analgesics in women was two times higher than men (p:0.052). The prevalence of pain medications taken in the last two weeks was 51.5% of all.

**Table-I:** Evaluation of IAU with demographic data and pain medication for Beers 2019 and STOPP/START criteria Version-2.

Variable	Classification	N (%)	IAU by BEERS P Value	IAU by STOPP P Value	Underuse by START P Value
Age (years)	65-74 (young-old)	199 (%60.1)	0.088	0.982	0.467
	75-84 (old)	101 (%30.5)			
	≥85 (old-old)	31 (%9.4)			
Sex	Female	176 (%53.2)	0.536	0.499	0.05
	Male	155 (%46.8)			
Marital status	Married	197 (%59.5)	0.767	0.711	0.791
	Single	134 (%40.5)			
Education	Illiterate	105 (%31.7)	0.582	0.025*	0.117
	Primary or secondary school graduates	209 (%63.2)			
	High school or University graduates	17 (%5.1)			
Number of drug consumption	< 5 drugs	66 (%12.7)	0.15	0.03*	0.03*
	≥ 5 drugs (polypharmacy)	265 (%87.3)			

* P value <0.05.

The presence of pain in patients suffering from pain in the last three months was 90% (n:298). Pain prevalence in women was higher than men, significantly (p:0.014), but there was no gender difference in pain medication use (p:0.434). 25.4% of participants described their intensity of pain as mild pain (0-3 on a scale of 0-10), 65.9% had moderate pain (4-6 on a scale of 0-10), and 8.8% had severe pain (7-10 on a scale of 0-10). 37.2% of cases were recently diagnosed as chronic pain. IAU was higher in patients with chronic pain than those without. The most painful region was lower extremity (knee) (26.9%), but the most frequently used IAU region was lower back (20.1%). The presence of headache, pain in the upper back, and pain in other regions were 21.8%, 7.9%, and 12.4%, respectively. There was a difference between painful regions for IAU (Beers P-value<0.001 STOPP P-value=0.05 START P-value<0.001). Participants had a 35.6% of pain every day, 14.8% of pain once/twice a week, 21.1% of pain once/twice a month, 18.4% of pain once/twice a year while 10% of all had never pain complaint.

**Fig.1:**
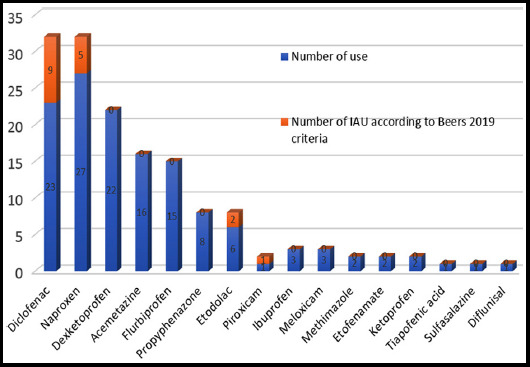
Evaluation of NSAIDs use according to Beers criteria

About 51.1% of participants were taking non-opioid analgesics. The prevalence of NSAID use in those taking nonopioids was 8.2% for naproxen, 6.9% for diclofenac, and 23.9% for paracetamol. The most commonly used analgesic drug was naproxen (8.2%), while the most common cause of IAU was diclofenac (2.7%). 39% of diclofenac use, 18.5% of naproxen use, and 33% of etodolac use were IAU. Fig.1 shows the evaluation of NSAIDs use according to Beers criteria.

Only paracetamol use was found to be 23.9%, while both paracetamol and NSAID users were 22.7%. The frequency of multiple NSAID molecules’ use was 13%. The prevalence of any NSAID use was 33.2% of all. 20.2% (n: 67) of participants were using only one type of NSAID, 10.3% (n: 34) using two types of NSAIDs and 2.7% (n: 9) three types of NSAIDs.

IAU prevalence detected by STOPP Version-2 was higher than that of the Beers 2019 criteria (19.6%, 14.5%, respectively; P<0.04; Z=–2.5) with a moderate agreement in between (kappa=0.458). IAU was found to be 15.4% according to START criteria. The underuse of analgesics was higher than inappropriate use. IAU presence determined by STOPP criteria but not determined according to Beers criteria was 10.3% of cases.

Most commonly IAU reason for Beers criteria was; ‘’Use with caution in patients with heart failure who are asymptomatic; avoid in patients with symptomatic heart failure: NSAIDs and COX-2 inhibitors (n:22, 6.6%)’’. Accordingly, the most common opposed STOPP criteria for IAU was ‘’ C11. NSAID with concurrent antiplatelet agent(s) without PPI prophylaxis (increased risk of peptic ulcer disease) ‘(n:28, 8.5%) whereas it was ‘’H1- High-potency opioids in moderate-severe pain, where paracetamol, NSAIDs or low-potency opioids are not appropriate to the pain severity or have been ineffective’’ (n:51, 15.4%) with the START criteria as underused.

**Fig.2:**
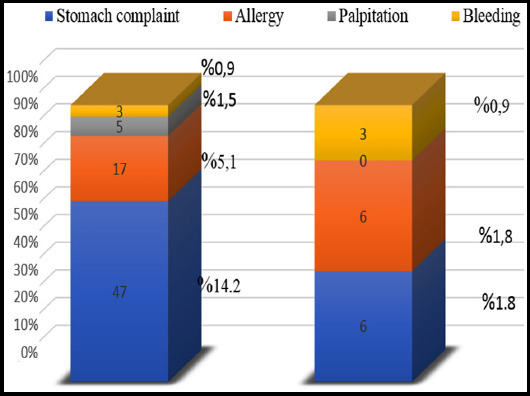
Evaluation of NSAIDs’ side effects and admission to emergency department

21.8% of the participants stated that they had side effects related to painkillers (stomach complaints, 14.2%, allergy 5.1%, palpitations 1.5%, bleeding 0.9%), as seen in Fig.2. The frequency of admission to the emergency department due to side effects was 4.5%, and palpitation was not obtained in any cases.

40.2% of the patients had no awareness about painkillers and side effects. Patients had been informed; 44.4% from doctors/health professionals, 11.2% from acquaintances, 3% from pharmacists, 1.2% from multi-media.

### Correlations

Weak and medium correlations were found between the IAU and *number of drugs* (Beers r=0.169 p=0.02, STOPP r=0.210 p<0.001, START r=0.106 p=0.05); *number of disease* (Beers r=0.134 p=0.014, STOPP r=0.77 p=0.02, START p=0.01 r=0.999); *pain score* (Beers r=0.149 p=0.007, STOPP r=0.197 p<0.001, START r=0.282 p<0.001). There was no significant difference between IAU and age or number of diseases (p>0.05).

### Logistic Regression Analysis

Factors associated with IAU were analyzed for the Beers, STOPP, and START criteria. In the analysis number of chronic diseases is not a significant predictor for all criteria. The risk factors associated with IAU emerged as a higher drug number and a higher pain score. Table-II shows the evaluation of risk factors affecting IAU by logistic regression analysis.

**Table-II:** Evaluation of independent risk factors effective in IAU by logistic regression analyses.

Criteria	Independent Risk Factors	P Value	OR	95% CI	
Beers	Number of chronic diseases	0.485	0.909	0,696	1.188
	Number of drugs	0.012*	1.214	1.044	1.411
	Pain score (VAS)	0.011*	1.289	1.061	1.568
Stopp	Number of chronic diseases	0.840	0.976	0.768	1.239
	Number of drugs	0.007*	1.206	1.051	1.384
	Pain score (VAS)	0.001*	1.338	1.121	1.597
Start	Number of chronic diseases	0.180	0.829	0.631	1.090
	Number of drugs	0.028*	1.185	1.019	1.379
	Pain score (VAS)	<0.001*	1.762	1.377	2.256

* P value <0.05

## DISCUSSION

In this present study, elderly adults who applied to a tertiary hospital were evaluated for rational analgesic use. The presence of inappropriate NSAID use was found as high as the presence of inadequate use of opioids. Half of the elderly patients in our study use at least one analgesic, and at least one out of three patients use NSAIDs. The knowledge about analgesics was not enough in patients, although analgesics might have many side effects on cardiovascular, GIS, and other systems in geriatric physiology.

The presence of NSAID use in order were reported 20.3% from Germany (Inappropriate medication in patients with renal insufficiency in nursing homes), 25% from America (African Americans aged 65 and older from 16 churches located in south Los Angeles), 28% from Serbia (In total 20 nursing homes located in Belgrade).^13^-^15^ Because of the risk for lack of data, we compared both drug monitoring systems and answers to the survey. The patients’ answers showed analgesic use in 49.8% of cases, but the electronic data findings in our study obtained 51.5% analgesic (33.2% NSAID ) use rate. It shows that patients tend not to mention their analgesic use. However, 33.2% is a critical NSAID usage value for elderly patients, even if the prevalence of pain is high (90%).

The IAU prevalence was detected as 14.5% by Beers 2019 criteria. Among patients admitted to a health center, the IAU prevalence varied widely being as low as 2.5% as in tertiary care teaching centres by “Beers 2012” from India, 7.1% from France in geriatric units in 28 hospitals by “Delphi process containing Beers 2015”, 9.3% from Turkey in a geriatric clinic of a university hospital by “Beers 2012” and as high as 43.04% among older Koreans by “Beers 2012” from Korea.^16^-^18^

Non-steroid anti-inflammatory drugs (NSAIDs) are largely used in pain management, even for long periods (months or years). While gastrointestinal side effects such as bleedings from peptic ulcers are known, the cardiovascular side effects have received much less attention. A warning (Nota 66) on the careful prescription of NSAIDs in patients with overt heart disease, such as coronary artery disease and heart failure, was published by the Italian Drug Agency a few years ago.^19^ In our study, naproxen was the most commonly taken NSAID, but diclofenac was the most common reason for IAU.

The IAU prevalence was defined as 19.6% by STOPP criteria Version-2. A study from the Netherlands (collected data of 182,000 patients of 49 general practitioners (GPs) gathered in the GPs’ database of the Academic Medical Center of Amsterdam) suggested that of patients with heart failure, 11.8% received ≥1 prescription for NSAIDs in 1 year, which is contraindicated.^20^ In our study, the most common criterion was using NSAID and antiplatelet medication together without PPI (8.5%). The second criterion was using NSAID with heart failure/ severe hypertension (4.5%). As a study from Korea, co-prescriptions prone to potentially harmful drug-drug interactions specified in the Beers Criteria 2015 were found NSAID, and corticosteroids use was 20.64% in cases.^21^ In our study, ıt was only 0.9% of cases.

The underuse of analgesic was detected as 15.4% by START criteria. That means opiate use is inadequate depending on WHO (World Health Organization) analgesic ladder. A study from the Netherlands using STOPP/START reported 12.9% a prescription for opiates^20^ In our study, opiate use was 4.8% in cases, but underuse was 15.4%. START IAU number was higher than the STOPP IAU number. It is highly probable that physicians are a little hesitant to prescribe opiates due to safety concerns.

A recent review of deaths from opioids concluded that the burden of opioid overdose in elderly adults requires special attention, noting the largest relative increase in opioids occurred in persons 55 to 64 (754% increase from 0.2% to 1.7%) and 65 years and older (635% increase from 0.01% to 0.07%) and the absolute number of deaths in this group is moderate. In 2016, 18.4% (7762 of 42 245) of all opioid-related deaths in the United States occurred among those aged 55 years and older.^22^,^23^

On the other hand, Beers’ list has two significant limitations, i.e., the focus on the American drug market and the restriction to potentially inappropriate medications. Pain medication list in Beers criteria did not include our patients using dexketoprofen, flurbiprofen, sulfasalazine, acemetacin, etofenamate, methimazole. Therefore in our opinion, Beers criteria are not truly reflecting the prevalence of inappropriate use of analgesics in Turkey.

We compared the success of Beers 2019 against STOPP Version-2 and found that the last updates on Beers criteria were not as good as STOPP Version-2 criteria yet.

### Limitation of the study

Our study was performed cross-sectionally and evaluated five months. The results of cross-sectional studies could, therefore, be biased due to seasonal or epidemical variations in medication use or the occurrence of pain complaints. Another limitation of our study was that OTC (over the counter) products might be procured without a prescription and the records of these drugs cannot be tracked from the monitoring systems, and records could not be included in the study.

## CONCLUSION

We conclude that STOPP screening criteria were more helpful than Beers criteria to reduce the negative consequences of analgesics, because of the significant difference was found between the two tests in terms of IAU cases. Inappropriate diclofenac use in the elderly patient was the well-marked finding of this present study, although NSAIDs have the cardiovascular or GIS side effects. The number of drug consumption and pain score (VAS) was predictors for IAU. The physician who may approach the elderly patient holistically and make the conventional follow-up of all treatments is the family physician in the first step. So, primary care is indispensable to control rational analgesic use under specific criteria in cases of pain management and polypharmacy.

### Authors Contribution

**MMB:** Did data collection, manuscript writing, editing, statistical analysis.

**OB:** Did data collection, editing of manuscript, review and final approval of the manuscript.
